# A Dive Into Yeast's Sugar Diet—Comparing the Metabolic Response of Glucose, Fructose, Sucrose, and Maltose Under Dynamic Feast/Famine Conditions

**DOI:** 10.1002/bit.28935

**Published:** 2025-01-26

**Authors:** Koen Johannes Anthonius Verhagen, Ilse Henrike Pardijs, Hendrik Matthijs van Klaveren, Sebastian Aljoscha Wahl

**Affiliations:** ^1^ Department of Biotechnology, Faculty of Applied Sciences Delft University of Technology Delft The Netherlands; ^2^ Lehrstuhl für Bioverfahrenstechnik Friedrich‐Alexander‐Universität Erlangen Germany

## Abstract

Microbes experience dynamic conditions in natural habitats as well as in engineered environments, such as large‐scale bioreactors, which exhibit increased mixing times and inhomogeneities. While single perturbations have been studied for several organisms and substrates, the impact of recurring short‐term perturbations remains largely unknown. In this study, we investigated the response of *Saccharomyces cerevisiae* to repetitive gradients of four different sugars: glucose, fructose, sucrose, and maltose. Due to different transport mechanisms and metabolic routes, nonglucose sugars lead to varied intracellular responses. To characterize the impact of the carbon sources and the dynamic substrate gradients, we applied both steady‐state and dynamic cultivation conditions, comparing the physiology, intracellular metabolome, and proteome. For maltose, the repeated concentration gradients led to a significant decrease in biomass yield. Under glucose, fructose, and sucrose conditions, *S. cerevisiae* maintained the biomass yield observed under steady‐state conditions. Although the physiology was very similar across the different sugars, the intracellular metabolome and proteome were clearly differentiated. Notably, the concentration of upper glycolytic enzymes decreased for glucose and maltose (up to −60% and −40%, respectively), while an increase was observed for sucrose and fructose when exposed to gradients. Nevertheless, for all sugar gradient conditions, a stable energy charge was maintained, ranging between 0.78 and 0.89. This response to maltose is particularly distinct compared to previous single‐substrate pulse experiments or limitation to excess shifts, which led to maltose‐accelerated death in earlier studies. At the same time, enzymes of lower glycolysis were elevated. Interestingly, common stress‐related proteins (GO term: cellular response to oxidative stress) decreased during dynamic conditions.

## 1 Introduction

Microbes experience dynamic conditions in natural habitats, as well as in engineered environments, particularly in large‐scale bioreactors that exhibit increased mixing times. With incomplete mixing, inhomogeneous zones arise, characterized by variations in oxygen and substrate concentrations (Haringa et al. 2016) are observed. Especially under carbon‐limited feeding conditions, which are necessary to avoid by‐product formation in industrial hosts like *Saccharomyces cerevisiae*, significant gradients in substrate concentration can be expected (Suarez‐Mendez et al. 2014). Such short and dynamic conditions require microbes to adjust their metabolism. One approach to generate reproducible dynamic conditions is the application of a block‐wise feeding regime, leading to repetitive cycles of feast and famine (Suarez‐Mendez et al. 2014).

Suarez‐Mendez et al. (2014) demonstrated that there are several adaptations of *S. cerevisiae* metabolism to repetitive glucose concentration gradients. Here, we expand this approach to include different industrially relevant carbon sources. In addition to glucose, several other sugars play a dominant role in biotechnology, with maltose being a widely available carbon source from grains, and sucrose primarily derived from sugar cane. Compared to glucose, these substrates exhibit different transport mechanisms and distinct signaling pathways (Lagunas 1993). Given these differences, glycolysis is expected to be perturbed in various ways by the different sugars: on the one hand, the intracellular substrate stimulus differs as transport kinetics are different, on the other hand differential regulatory expression of glycolytic enzymes will play a crucial role. Various studies have explored the effects of substrate pulses on carbon‐limited *S. cerevisiae* cultures, including those with maltose (Postma et al. 1990), fructose (Bosch et al. 2008), and xylose (Borgström et al. 2019). However, these studies did not consider the repetitive dynamic conditions observed in the context of industrial scale‐down.

**Figure 1:**
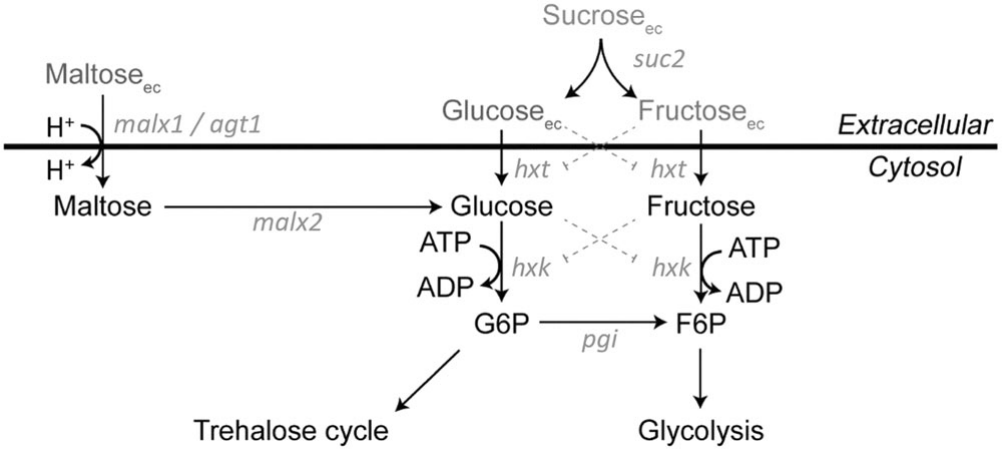
Schematic overview of the uptake of glucose, fructose, and maltose, as well as the extracellular hydrolysis of sucrose in *Saccharomyces cerevisiae*, coupled to reactions and pathways in central carbon metabolism. Here malx1 represents mal11, mal21, mal31, mal41, and mal61; malx2 represents mal12, mal22, mal32, mal42, and mal62. Reported substrate inhibition on enzyme activities is shown with dotted lines. *Source:* Adapted from (Lao‐Martil 2022).

Sucrose is known to be degraded extracellularly into glucose and fructose, catalyzed by invertase (Bowski et al. 1971) (see Figure 1). The invertase enzyme, encoded by *SUC2*, can exist in two forms: A glycosylated variant located in the periplasmic space, hydrolyzing extracellular sucrose into glucose and fructose, and a nonglycosylated version that remains in the cytoplasm of the cell.

The two monosaccharides, glucose and fructose, are very similar substrates (both C_6_H_12_O_6_). Furthermore, both enter the cell via one of the Hxt transporters, with Hxt1, Hxt3, and Hxt7 being the most relevant (Berthels et al. 2004; Guillaume et al. 2007; Özcan and Johnston 1999). Intracellularly, both glucose and fructose are further metabolized through phosphorylation by hexokinase to G6P and F6P, respectively. For both sugars, Hxk2 is the major paralog responsible for this reaction. Bisson and Fraenkel noted that glucose can also be converted by glucokinase (Glk1) (Bisson and Fraenkel 1983). Under low substrate conditions, they observed a high affinity for these sugars, with K_M_ = 1.5 mM and K_M_ = 6 mM for glucose and fructose, respectively. Consequently, slightly higher residual substrate concentrations were observed for *S. cerevisiae* grown in a chemostat on fructose compared to glucose, due to this difference in affinity; however, similar yields of biomass were obtained (Diderich et al. 1999). Under high substrate concentrations, a low‐affinity uptake system is active, with affinity constants of K_M_ = 20 mM for glucose and K_M_ = 40 mM for fructose.

Maltose is a disaccharide comprising two 1,4‐α‐linked glucose molecules (Barnett 1981). In contrast to glucose and fructose, maltose is transported via an active transport mechanism, specifically the maltose‐proton symporter. Once inside the cell, maltose is hydrolyzed into two glucose molecules by α‐glucosidase, also known as “maltase” (Han et al. 1995; Serrano 1977) (see Figure 1). The proton symport results in a net loss of one ATP per mol of maltose due to the proton export for pH homeostasis. Compared to glucose, this results in a decrease of the ATP gain by 0.5 ATP per C6 unit. Weusthuis et al. (Weusthuis et al. 1993) reported a 25% decrease in biomass yield of *S. cerevisiae* when grown anaerobically on maltose compared to glucose; however, aerobically, this effect will be much smaller (Rich 2003).

*S. cerevisiae* has been reported to be (hyper)sensitive to sudden changes in extracellular maltose concentration (Postma et al. 1990). This phenomenon, commonly referred to as ‘maltose accelerated death’, occurs due to the unrestricted uptake of maltose, leading to the accumulation of glucose and protons, which ultimately results in cell death (Van Urk et al. 1988). While this effect was initially observed for extracellular maltose concentrations exceeding 50 mM, it is important to note that the K_M_ for maltose transport is 2.5 mM (Postma et al. 1990), and already lower concentrations may already impact the energy homeostasis of the cell. Additionally, the symport mechanism, which transports one proton per maltose reduces the ATP gain from maltose compared to glucose, as the excretion of the proton consumes one ATP.

## 2 Regulatory Mechanisms in *S. cerevisiae*

Metabolism is regulated at various cellular levels. Allosteric activation or inhibition of the enzyme reaction and post‐translational modifications are rapid mechanisms that can respond to sudden environmental changes within seconds. In contrast, adjustments to enzyme concentrations through gene expression (regulation) occur on a timescale of hours (Belinchón and Gancedo 2007b; Broach 2012; Conrad et al. 2014; Peeters and Thevelein 2014; Rolland, Winderickx, and Thevelein 2002; Santangelo 2006). In these regulatory pathways, the sugars serve both as substrates and signaling molecules. Glucose, in particular, is the preferred substrate in yeast, controlling not only its utilization, but also the consumption of other carbon sources. This phenomenon, known as catabolite repression, leads to the preferential consumption of glucose over other available saccharides such as sucrose, fructose, or maltose (Belinchón and Gancedo 2007b, 2007a; Conrad et al. 2014; Gancedo 1998; Santangelo 2006).

In addition to catabolite repression, cell growth in *S. cerevisiae* strains is regulated by glucose signaling through protein kinase A (PKA). Specifically, PKA is known to regulate the expression of proteins involved in biomass synthesis, such as ribosomes (Broach 2012; Conrad et al. 2014; Peeters et al. 2017; Santangelo 2006). A major signaling cascade that activates PKA is controlled by cyclic AMP (cAMP), alongside the Gpr1/Gpa2 and Ras proteins (Broach 2012; Conrad et al. 2014; Cullen and Sprague 2012; Rolland, Winderickx, and Thevelein 2002). The Gpr1/Gpa2 signaling system is stimulated by various substrates, which in turn, affect the activity of adenylyl cyclase. Given the differing affinities of these proteins for each sugar, distinct responses are anticipated. Notably, the system exhibits a higher affinity for sucrose compared to glucose, while it is far less sensitive to fructose, that mainly activates PKA activity via the Ras1/Ras2 branch instead (Lemaire et al. 2004; Peeters et al. 2017).

The signaling response of cAMP in *S. cerevisiae* upon switching from ethanol to different sugar carbon sources revealed a similar response for glucose and sucrose; however, no significant cAMP response was observed for maltose. For fructose, a lower cAMP response—approximately half the magnitude of that observed for glucose and sucrose—was observed (Botman et al. 2021). This suggests that these different sugar substrates elicit markedly different responses compared to each other. Nevertheless, the precise mechanism linking this signaling cascade to cell growth in response to varying sugar levels remains unidentified (Broach 2012; Conrad et al. 2014). Additionally, CEN. PK113‐7D, the strain used in this study, has a mutation in the CYR1 gene, which encodes a key enzyme involved in cAMP production, and cAMP‐dependent protein kinase signaling (Nijkamp et al. 2012). Consequently, this this strain does not exhibit a cAMP peak following sudden exposure to high glucose concentrations. Botman et al. (2024) further note that little difference in the various components of the cAMP signaling cascade was observed during steady‐state growth with the CYR1 mutant strain, which was also observed in other studies (Kümmel et al. 2010). However, upon transferring an ethanol‐grown culture to 10 mM sucrose, a 6% decrease in growth rate was noted for the CYR1 mutant compared to the wild‐type strain. This indicates that the mechanism of PKA activation in CEN. PK113‐7D differs from strains not deficient in CYR1, yet the exact mechanism through which PKA is activated in this strain remains unknown.

In this study, we compare the physiology of *S. cerevisiae* CEN. PK113‐7D under different feeding regimes and carbon substrates. Additionally, we compare the metabolome and proteome across these conditions to gain insights into potential adaptations.

## 3 Results and Discussion

All cellular responses were compared concerning differences in feeding conditions (continuous vs. block‐wise) and the used carbon sources. The results are structured by analyzing (1) the extracellular environment, (2) the phenotype, and then (3) metabolic and proteomic responses. Note that all cultures and conditions were carried out with the same amount of substrate (in terms of moles of carbon) and water per time interval, resulting in a comparable growth rates across all feeding conditions and carbon sources. In this study, metabolomics datasets were generated for fructose, sucrose, and maltose, which was subsequently compared with a glucose metabolomics dataset previously generated by Suarez‐Mendez et al. (2014) using the same experimental approach and setup.

### 3.1 Substrates in the Extracellular Environment

**Figure 2:**
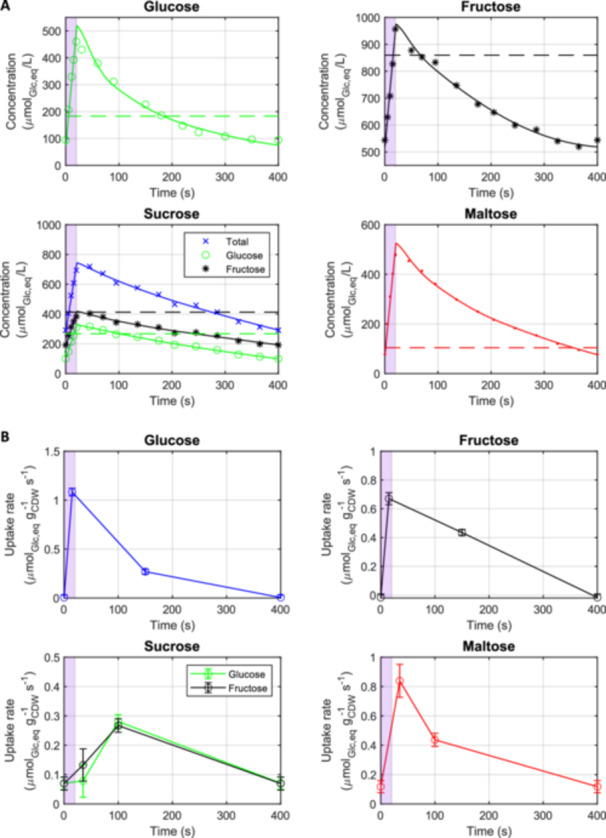
Measured extracellular sugar concentrations (A) and estimated sugar uptake rates in µmol/g_cdw_/s (in glucose equivalents, that is, 1 mol maltose = 2 mol glucose equivalents) (B) during the 400 s cycle across four different cultivation conditions: glucose, fructose, sucrose, and maltose as substrates. Data for the glucose cultivation conditions were generated by Suarez‐Mendez et al. (2014), while data for the fructose, sucrose, and maltose cultivation conditions were generated in this study. For all conditions, the same amount of carbon was supplied. The estimation of the uptake rates is based on a piece‐wise linear function approximation taking into account the biomass concentration. Due to the cyclic nature of the repetitive feast/famine regime, the first point of the cycle at 0 s corresponds to the last point at 400 s. Measurements were performed after five residence times, approximately 50 h after initiation of the block‐wise feeding. Dashed lines indicate residual substrate concentrations observed during steady‐state cultivation.

The extracellular sugar concentrations were measured under both steady‐state and feast/famine (FF) conditions after five residence times, allowing the cells to adapt to the imposed feeding regime (see Figure 2). For the monosaccharides, a significant difference was observed: With fructose, a higher residual sugar concentration was measured under both the steady‐state (Frc/Glc = 4.7 fold) and the FF (Frc/Glc = 5.8 fold) condition (concentrations taken at end of cycle). This difference in residual sugar concentration can be attributed to the differences in the affinities of hexose transporters. For glucose transport, a higher affinity (K_M_ = 1.5 mM) was reported compared to fructose (K_M_ = 6 mM) (Bisson and Fraenkel 1983). Due to the higher K_M_ for fructose, the relative changes in concentration within the feast famine cycle were smaller under fructose conditions (max/min Frc = 1.8 compared to max/min Glc = 4.9). Consequently, a less dynamic uptake rate and “milder” intracellular metabolic response during the FF cycle is anticipated.

With sucrose as the substrate, we observed that the fed sucrose was immediately converted into glucose and fructose by invertase, resulting in a residual sucrose concentration of less than 0.01 mM. Compared to the respective monosaccharide cultivations, the difference in residual sugar concentration was smaller: At steady‐state, the residual fructose concentration was 1.5‐fold higher than that of glucose (compared to Frc/Glc = 4.7 for the reference). Under FF conditions, the residual glucose concentration at the end of the cycle with sucrose feeding was very similar to that observed with glucose as the sole substrate. In contrast to this observation, the residual fructose concentration was significantly lower compared to the fructose‐only condition. Under the sucrose condition, the uptake rate for each sugar was halved compared to the respective monosaccharide conditions, and a decrease in the rate commonly requires lower residual concentrations. Nevertheless, with the residual only lower for fructose suggests that there is a sucrose specific regulation mechanism or cotransport effects.

**Figure 3:**
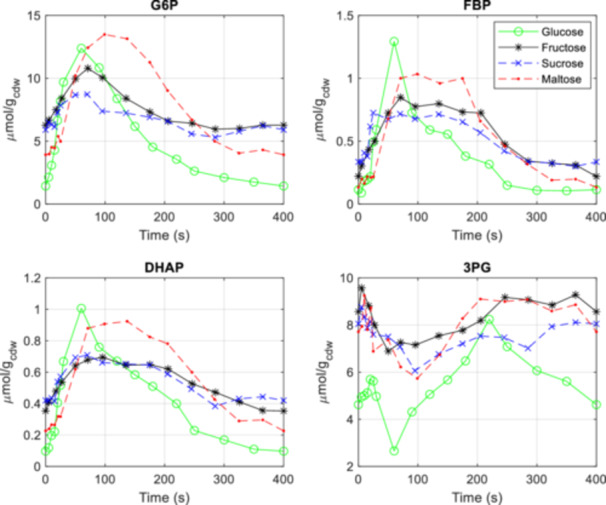
Concentration measurements of intracellular glycolytic metabolites during the feast/famine cycle for glucose (green), fructose (black), sucrose (blue), and maltose (red) as substrates. Dashed lines represend disaccharide sugars, while solid lines are used for monosaccharide sugars. Data for the glucose cultivation conditions were taken from Suarez‐Mendez et al. (2014), whereas the data for the fructose, sucrose, and maltose were generated in this study.

With maltose as the substrate, no extracellular hydrolysis was observed; that is, no glucose was found in the extracellular space. Instead, the extracellular maltose concentration followed a pattern similar to that of the glucose conditions. Furthermore, similar to the glucose conditions, a higher affinity for maltose under FF compared to the steady‐state condition was found. Comparable (glucose equivalent) residual sugar concentrations were measured under both steady state (Mal_glc, eq_/Glc = 1.1) and FF (Mal/Glc = 0.8) conditions. However, relative changes were slightly larger under maltose conditions (max/min Mal = 6.2 compared to max/min Glc = 4.9). Notably, a faster decrease in glucose concentration was observed during FF cycles, with the substrate uptake reaching zero at the end of the cycle (Figure 3), while maltose uptake was still active.

### 3.2 Uptake Rate Estimation

While steady‐state uptake rates were very comparable among the different sugars (see Table 1), a different trend is observed for the dynamic uptake rates during the FF cycles (Figure 2). To quantitatively compare the transport of sugars into the cell under FF conditions, a piecewise affine (PWA) rate approximation was calculated based on the concentration measurements. The time points at 0, 15, 150, and 400 s were used as breakpoints (Schumacher and Wahl 2015; Vieth 1989) for the monosaccharides and 0, 35, 100, and 400 s for disaccharides. Furthermore, the first and last time points were coupled to reflect the cyclic nature of the imposed feeding regime (see Figure 2). Various combinations of breakpoints were manually tested, and the above ones were selected as these combinations resulted in the lowest sum of residual squares (SSR).

**Table 1:** Reconciled average biomass‐specific rates and yields for steady‐state and feast/famine conditions using different sugars as substrates. Data for the glucose cultivation conditions were obtained from Suarez‐Mendez et al. (2014), while data for the fructose, sucrose, and maltose cultivation conditions were generated in this study. Standard deviations are calculated from three technical replicates for the fructose, sucrose, and maltose dataset. For the glucose dataset, standard deviations were calculated from two technical replicates. The relative changes between steady‐state and feast/famine conditions are given in the last column.

Glucose			
Rate/yield	Steady‐state	Feast/famine (averaged over cycle)	Change (%)
*−q*_*s*_ *(mCmol · g*_*X*_^*‐1*^ *· h* ^*‐1*^ *)*	6.89 ± 0.21	7.06 ± 0.07	2
*µ (h* ^*‐1*^ *)*	0.101 ± 0.0002	0.100 ± 0.0006	0
*−q*_*O2*_ *(mmol · g*_*X*_^*‐1*^ *· h* ^*‐1*^ *)*	2.67 ± 0.21	2.70 ± 0.04	1
*q*_*CO2*_ *(mCmol · g*_*X*_^*‐1*^ *· h* ^*‐1*^ *)*	2.85 ± 0.21	2.80 ± 0.05	−2
*C*_*X*_ *(g*_*X*_ *·L* ^*‐1*^ *)*	3.64 ± 0.16	3.46 ± 0.17	−4
*Y*_*X/S*_ *(g*_*X*_ */g*_*S*_ *)*	0.49	0.47	−3

Fructose			
Rate/yield	Steady‐state	Feast/famine (averaged over cycle)	Change (%)
*−q*_*s*_ *(mCmol · g*_*X*_^*‐1*^ *· h* ^*‐1*^ *)*	6.84 ± 0.12	7.07 ± 0.18	3
*µ (h* ^*‐1*^ *)*	0.104 ± 0.0007	0.098 ± 0.0009	−6
*−q*_*O2*_ *(mmol · g*_*X*_^*‐1*^ *· h* ^*‐1*^ *)*	2.75 ± 0.14	2.58 ± 0.16	−6
*q*_*CO2*_ *(mCmol · g*_*X*_^*‐1*^ *· h* ^*‐1*^ *)*	3.12 ± 0.09	3.01 ± 0.15	−4
*C*_*X*_ *(g*_*X*_ *·L* ^*‐1*^ *)*	3.63 ± 0.07	3.51 ± 0.04	−3
*Y*_*X/S*_ *(g*_*X*_ */g*_*S*_ *)*	0.49	0.47	−3

Sucrose			
Rate/yield	Steady‐state	Feast/famine (averaged over cycle)	Change (%)
*−q*_*s*_ *(mCmol · g*_*X*_^*‐1*^ *· h* ^*‐1*^ *)*	7.00 ± 0.09	7.23 ± 0.22	3
*µ (h* ^*‐1*^ *)*	0.101 ± 0.0004	0.100 ± 0.0003	0
*−q*_*O2*_ *(mmol · g*_*X*_^*‐1*^ *· h* ^*‐1*^ *)*	2.77 ± 0.14	2.81 ± 0.10	1
*q*_*CO2*_ *(mCmol · g*_*X*_^*‐1*^ *· h* ^*‐1*^ *)*	3.05 ± 0.13	3.11 ± 0.08	2
*C*_*X*_ *(g*_*X*_ *·L* ^*‐1*^ *)*	3.55 ± 0.11	3.45 ± 0.02	−3
*Y*_*X/S*_ *(g*_*X*_ */g*_*S*_ *)*	0.50	0.48	−3

Maltose			
Rate/yield	Steady‐state	Feast/famine (averaged over cycle)	Change (%)
*−q*_*s*_ *(mCmol · g*_*X*_^*‐1*^ *· h* ^*‐1*^ *)*	6.99 ± 0.06	7.61 ± 0.04	9
*µ (h* ^*‐1*^ *)*	0.099 ± 0.0008	0.100 ± 0.0005	0
*−q*_*O2*_ *(mmol · g*_*X*_^*‐1*^ *· h* ^*‐1*^ *)*	2.87 ± 0.13	3.24 ± 0.08	13
*q*_*CO2*_ *(mCmol · g*_*X*_^*‐1*^ *· h* ^*‐1*^ *)*	3.12 ± 0.12	3.58 ± 0.11	15
*C*_*X*_ *(g*_*X*_ *·L* ^*‐1*^ *)*	3.57 ± 0.07	3.27 ± 0.03	−8
*Y*_*X/S*_ *(g*_*X*_ */g*_*S*_ *)*	0.48	0.44	−8

Comparable to the concentration measurements, clear differences in the uptake rate dynamics were observed for the monosaccharides. As expected, due to the lower affinity (higher K_M_), the dyanamic of fructose uptake rate was less pronounced compared to that of glucose during the cycle. The maximum specific uptake rate for glucose was 1.08 µmol_glc_/g_cdw_/s, compared to 0.68 µmol_frc_/g_cdw_/s for fructose, nearly two‐fold lower. Canelas et al. (2011) observed a switch to respiro‐fermentative metabolism at around 1 µmol_glc_/g_cdw_/s under steady‐state conditions, suggesting there could be a short period with overflow metabolism. Nevertheless, no ethanol was detected in the extracellular samples at any time point, indicating fully aerobic metabolism throughout the entire cycle for all carbon sources.

Under sucrose conditions, a very high invertase rate was observed. The rate of invertase exceeded the respective sugar uptake rates, resulting in no residual sucrose being measured at any time point during the cycle, while glucose and fructose accumulated in the initial seconds. The total monosaccharide uptake rates peaked at 0.55 µmol_glc, eq_/g_cdw_/s, dropping to 0.14 µmol_glc, eq_/g_cdw_/s by the end of the cycle. Thus, although the high invertase activity essentially led to a “two monosaccharide” feeding regime, the uptake rates did not reflect those of glucose nor fructose feeding conditions. Especially, the uptake rate dropped to zero for the monomer feeding towards the end of the cycle, while a residual rate was maintained during sucrose conditions. Assuming a Monod‐type uptake kinetics, and with a halved feeding amount (compared to glucose and fructose alone), this suggests a significant change in affinity or uptake mechanism with inferences of glucose and fructose.

Surprisingly, the maltose uptake rate, which is catalyzed by an active proton symport mechanism, was lower compared to glucose uptake, which relies on facilitated transport. The maximum rate achieved was 0.85 µmol_glc, eq_/g_cdw_/s, representing only about 78% of the maximum glucose uptake rate. Additionally, maltose transport continued until the end of the cycle, maintaining a minimum uptake rate of 0.11 µmol_glc, eq_/g_cdw_/s with a residual concentration of approximately 100 µmol/L. In contrast to glucose, this residual was just below the steady‐state value, while for glucose the residual at the end was about half the steady‐state value.

### 3.3 Average Biomass Specific Rates and Yields

The average growth rate across all sugars was maintained by setting a dilution rate of 0.1 h^‐1^. The resulting rates and yields under steady‐state and FF conditions, averaged over the entire cycle, are summarized in Table 1. In the continuous feeding regime, only minor differences were observed among the various sugars.

When comparing continuous and block‐wise feeding, differences were observed. For glucose, a slight decrease in biomass yield of −3% was found, along with minor differences in the extracellular rates. Similarly, slight declines in biomass yields were observed under fructose and sucrose conditions. In contrast, for maltose a significant decrease in biomass yield (−8%) accompanied by a 13% increase in respiratory activity during FF compared to steady‐state conditions. This reduction in biomass yield, coupled with the increased respiration, suggests an increased ATP consumption attributed to futile cycling or a stress response.

Previous studies by Postma et al. indicated that cultures grown under maltose‐limited conditions were unable to cope with a pulse of maltose. This phenomenon, called maltose‐accelerated death, resulted in significant maltose accumulation within the cell due to the high transport capacity. The accumulated maltose was further converted into glucose at a high rate, leading to a high intracellular osmotic pressure due to glucose accumulation. Additionally, the influx of protons coupled to maltose transport contributed to acidification of the cytosol, leading to cell death (Postma et al. 1990). In the current study, no excessive maltose uptake was detected (see above). However, it is plausible that substrate cycling may have induced energetic losses.

### 3.4 Intracellular Metabolite Concentration Dynamics

Based on the observed sugar uptake rate profiles (Table 2), we anticipated differences in intracellular metabolite concentrations. To investigate this, we measured the metabolites involved in central carbon metabolism under both, the steady‐state and the FF conditions.

**Table 2:** Comparison of selected metabolic concentration properties under feast/famine conditions across different sugars. The data for glucose cultivation conditions were obtained from Suarez‐Mendez et al. (2014), whereas the data for the fructose, sucrose, and maltose cultivation conditions were generated in this study. “Max/min” refers to the ratio of the observed maximum over minimum concentration throughout the cycle. “Peak time” indicated the time point at which the highest concentration measurement was obtained. “Fold change average” is calculated based on the average value of all measurements during the cycle compared to the steady‐state value.

Metabolite	Max/min ratio	Time peak concentration (s)	Fold change average FF vs SS
Glucose			
Extracellular glucose	4.88	20	1.12
G6P	8.65	60	1.05
FBP	14.66	60	0.59
3PG	2.14	220	0.88
Energy charge	1.13	60	0.98
Fructose			
Extracellular fructose	1.76	20	0.79
G6P	1.72	71	0.57
FBP	3.86	71	0.62
3PG	1.34	5	1.04
Energy charge	1.07	50	1.03
Sucrose			
Extracellular total sugar/glucose/fructose	2.47/3.17/2.10	20	0.72/0.75/0.70
G6P	1.48	70	0.88
FBP	2.17	70	0.72
3PG	1.45	5	1.11
Energy charge	1.05	70	0.98
Maltose			
Extracellular maltose	6.23	20	1.16
G6P	3.44	99	1.05
FBP	7.73	99	0.59
3PG	1.61	10	0.88
Energy charge	1.15	71	0.97

### 3.5 Glycolysis and Trehalose Cycle

When utilizing glucose as the substrate, the concentrations of upper glycolytic metabolites, such as glucose‐6‐phosphate (G6P), exhibited a rapid increase, peaking approximately 60 s after the start of the cycle. This delay is significant when compared to the maximum extracellular glucose concentration, which was observed after 20 s (Suarez‐Mendez et al. 2014) (see Table 2 and Figure 3). In contrast, an inverse pattern was observed for lower glycolytic intermediates, such as 3‐phosphoglycerate (3PG). Following the start of the feeding, 3PG showed a modest increase during the first 20 s, followed by a sharp decline until 60 s, after which the concentration began to recover. This inverse behavior may be attributed to the allosteric activation of the last (rate‐limiting) step of the lower glycolysis, pyruvate kinase, by fructose‐1,6‐bisphosphate (FBP) (Jurica et al. 1998). Toward the end of the cycle, after 220 s, the 3PG concentration decreased again, likely due to the lower uptake rate, resulting in a reduced influx into the metabolite pool.

The comparison of perturbation magnitudes indicates that the concentrations of some intermediates exceed the fluctuations observed in the extracellular space. For example, under glucose conditions, some intermediates, such as fructose bisphosphate (FBP), exhibit an increase of up to 15‐fold (FBP), whereas the extracellular max/min is approximately four‐fold (Table 2). In comparison, the magnitude of perturbation of FBP observed for fructose is nearly four‐fold lower than that of glucose. This trend is also apparent in the trehalose cycle and the pentose phosphate pathway (see Supporting Information S1).

Similarly, for the disaccharide sucrose, which exhibits lower maximal uptake rates, resulted in less pronounced changes of glycolytic intermediates when compared to glucose feeding conditions. For example, glucose‐6‐phosphate (G6P) showed approximately a ~ 50% increase under sucrose, compared to a ~ 750% increase observed with glucose. This trend is also observed in the pentose phosphate pathway and the trehalose cycle (see Supporting Information S1). Interestingly, downstream of FBP in the lower glycolytic pathway, larger fluctuations in metabolite concentrations were measured.

**Figure 4:**
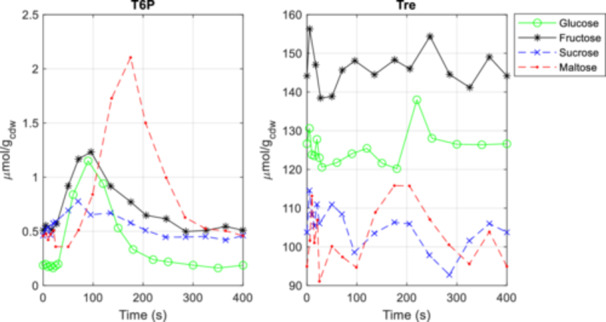
Concentration measurements of intracellular metabolites of the trehalose cycle during a 400 s feast/famine cycle for glucose (green), fructose (black), sucrose (blue), and maltose (red). Dashed lines indicate disaccharide sugars, full lines indicate monosaccharide sugars. Data for the glucose cultivation conditions was generated by Suarez‐Mendez et al. (2014), while data for the fructose, sucrose, and maltose cultivation conditions was generated in this study.

For maltose, a significant delay of 30 s in the metabolic responses can be observed compared to all other conditions (see also Supporting Information S1 for further metabolites). Additionally, a much higher peak was observed for trehalose‐6‐phosphate (T6P) (Figure 4), indicating a possible difference in the activity in the trehalose cycle under maltose conditions compared to glucose. T6P is reported to inhibit the glycolytic flux (Thevelein and Hohmann 1995), especially the enzyme HXK2. Here, the extracellular concentration was varying rapidly over the cycle and it is therefore not trivial to evaluate if there is an effect observed here. The potential increase in trehalose cycling could partly explain the observed decrease in biomass yield and increase in respiratory activity compared to glucose. Putative further mechanisms responsible for the yield decrease could be maltose cycling, especially between the cytosol and the extracellular space.

### 3.6 Energy Homeostasis–Nucleotides

**Figure 5:**
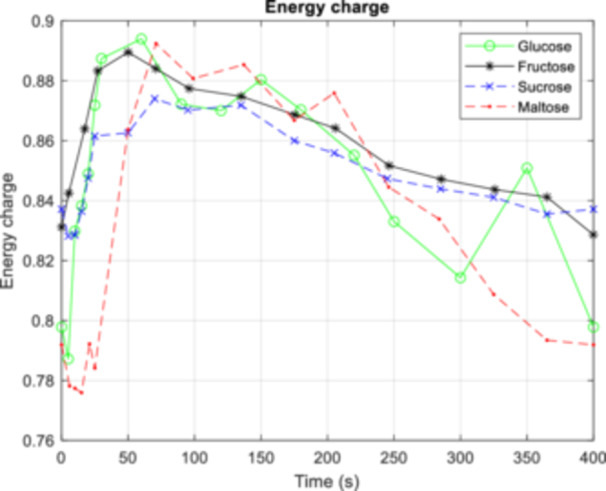
Energy charge (EC) during a 400 s feast/famine cycle for different substrates: glucose (green), fructose (black), sucrose (blue), and maltose (red). Dashed lines represent disaccharide sugars, while solid lines indicate monosaccharide sugars. Data for the glucose cultivation conditions were obtained from Suarez‐Mendez et al. (2014), whereas data for the fructose, sucrose, and maltose cultivation conditions were generated in this study.

In contrast to previous single pulse experiments, the conditions here did not result in the so‐called “ATP paradox,” which describes a decrease in energy charge and adenylate nucleotide levels despite the availability of increased substrate (Somsen et al. 2000; Walther et al. 2010). Under the repetitive cycles of limitation and excess in this study, cells adapted and achieved stable AxP levels (see Supporting Information Figure S2) across all sugars tested. Furthermore, we find nearly energy homeostasis, with an energy charge (Ball and Atkinson 1975) sustained between 0.7 and 0.89 throughout the FF cycle. Interestingly, again a significant delay was observed for maltose conditions, where the energy charge lagged approximately 30 s behind that of the other sugars (Figure 5).

### 3.7 Proteome Adaptations

Simulations based on kinetic models suggested that the change in metabolic response between steady‐state and FF conditions cannot not be solely attributed to metabolite and enzyme kinetics (Lao‐Martil et al. 2023). The model was only successfully fitted to the experimental data after additional adjustments to specific protein concentrations, especially the hexose transporter(s) and hexokinase/glucokinase. Consequently, we anticipated and measured changes in the proteome composition between steady‐state chemostat and dynamic FF conditions across the different sugar substrates were expected and measured.

### 3.8 Nontargeted Proteome Comparison

The proteomic comparison between conditions was performed using a label‐free quantification approach. In total, 1126 proteins from 4748 peptides were identified in technical duplicates, covering 18% of metabolic proteins (345 out of 1928) reported in the KEGG database (https://www.kegg.jp/kegg-bin/download_htext?htext=sce00001).

**Figure 6:**
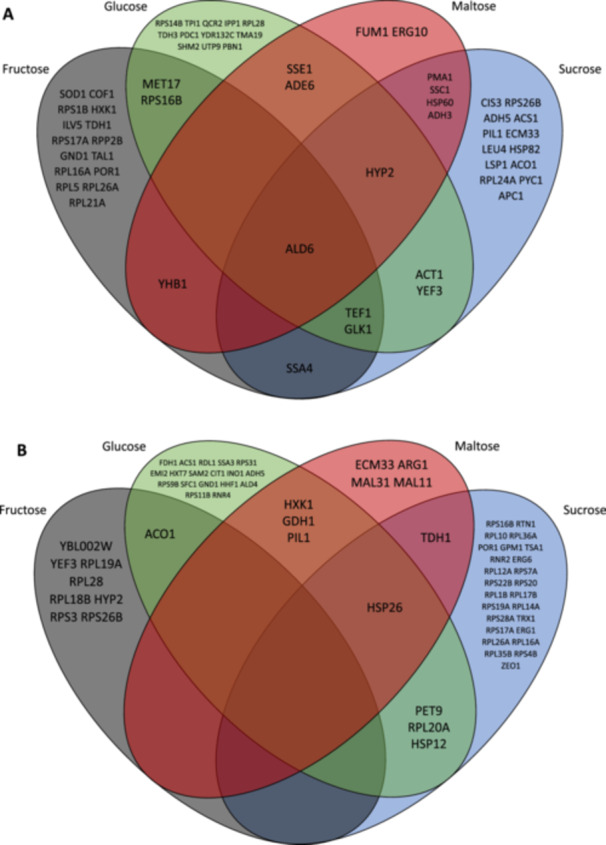
A venn diagram of upregulated proteins (A) and downregulated proteins (B) (> 0.25 fold change, > 0.02% of proteome) from steady state to feast/famine for all four substrates.

For the comparison of the different conditions, we assumed protein levels to be different if a fold change greater than 0.25 was observed, along with a minimum abundance of 0.02% of the total proteome was measured. For the initial comparison, steady‐state conditions were used as reference. Sucrose, fructose, and glucose were found to trigger most proteome changes, with 12 enzymes uniquely upregulated under glucose, 13 under sucrose, and 15 under fructose (Figure 6). In contrast, maltose exhibited only two uniquely differentially regulated proteins. Aldehyde dehydrogenase (Ald6p) was the only protein that was increased across all substrates. This enzyme facilitates the conversion of (accumulated) acetaldehyde to acetyl‐CoA under stress conditions (Aranda and del Olmo 2003). This may be associated with the increased acetate production observed under FF conditions as compared to steady‐state.

Under fructose and sucrose conditions, Ssa4p is upregulated (Figure 6). Ssa4p is a chaperone protein belonging to the *S. cerevisiae* SSA subfamily of cytosolic Hsp70 proteins. Hsp70 proteins function as molecular chaperones, binding to newly translated proteins to facilitate proper folding and prevent aggregation or misfolding (Mayer and Bukau 2005). Although knockout of Ssa4p resulted in a phenotype which was non‐distinguishable from that of the wild‐type, its expression has been shown to be linked to various stresses, including heat shock, cold, and ethanol stress. Furthermore, changes during the diauxic shift were observed (Boorstein and Craig 1990; Kandror et al. 2004; Quan et al. 2004; Werner‐Washburne et al. 1989). The observed increase in SSa4p levels under FF conditions for fructose and sucrose here is a new condition of upregulation.

Additionally, the common upregulated proteins across glucose, fructose, and sucrose conditions include Tef1p and Glk1p, which are an elongation factor and a paralogue of hexokinase (Hxk2p), respectively. The latter exhibits different kinetic properties, especially with respect to allosteric regulation by T6P (Blázquez et al. 1993).

An analysis of the decreased protein levels from steady‐state to FF conditions revealed substrate‐specific changes. There was no protein commonly decreased across all substrates (Figure 6). Under sucrose FF 31 proteins showed a decrease, while only nine proteins were downregulated using maltose as substrate. Downregulated for glucose, maltose and sucrose conditions, was Hsp26p, a molecular chaperone. Hsp26p is minimally expressed in unstressed cells but is strongly induced under various stress conditions, including carbon starvation (Ferreira et al. 2006, 26; Lien et al. 2019). These observations suggest that under FF conditions, on average, cells appear to experience less stress from carbon starvation compared to steady‐state conditions with constant limitation.

A common decrease in protein levels was observed for hexokinase I (Hxk1p), glutamate dehydrogenase I (Gdh1) and Pil1 for maltose and glucose conditions. The reduction in hexokinase activity was crucial in a previous modeling study for accurately reproducing the metabolite measurements within a kinetic model of *S. cerevisiae* central carbon metabolism (Lao‐Martil et al. 2023). The regulation of Gdh1 expression by the nature of the carbon sources has been described (DeLuna et al. 2001), but not yet by carbon source dynamics.

### 3.9 Pathway Enrichment Analysis

A pathway enrichment analysis was conducted using the gene ontology biological process terms (GO terms, which were downloaded from the gene ontology (22 October 2022) (The Gene Ontology Consortium 2023) (Ashburner et al. 2000). This analysis facilitates the identification of global changes in protein groups related to specific biological functions. The analysis was performed using a Fisher's exact test, selecting for proteins groups of more than five proteins of which at least 2/3 of proteins were significantly differentially expressed (Schessner, Voytik, and Bludau 2022). Here the *p*‐value (displayed as −log10[p]) indicates the significance of the pathway protein level changes, “n significant” indicates the number of significantly changed proteins. The “odds ratio” quantifies the ratio of the odds for observed changes over the odds of a random change. An odds ratio larger than 1 suggests that the observed changes are not random events but rather indicative of systematic changes in expression due to the differing conditions.

Steady‐state to FF: Across all substrate conditions, no significant changes (−log10 (*p*‐value) > 0.5) were found in specific biological functions. Consequently, although these conditions exhibit distinct metabolic responses, the observed changes appear to be primarily attributed to kinetics or post‐translational modifications, rather than pathway specific variation in protein concentrations.

**Figure 7:**
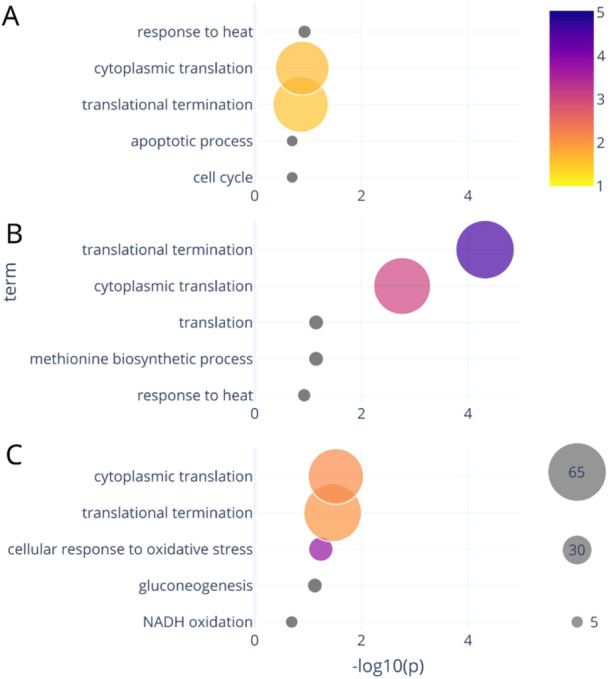
Pathway enrichment analysis (GO terms) based on (GO terms) based on Fisher's exact test for the feast/famine conditions. Compared are fructose (A), sucrose (B), and maltose (C) to glucose as reference. For all significant terms, the *p*‐value, group size, and the odds ratio are presented. The plots were generated using the visualization toolbox developed by Schessner, Voytik, and Bludau (2022).

Comparison between substrates: The GO analysis was the conducted to compare the FF proteomes across different substrates (Figure 7). Significant changes were observed in the categories “cytoplasmic translation” and “translational termination” for all other sugar conditions when compared to glucose. These GO terms were also observed earlier for CEN. PK113‐7D when comparing sucrose to glucose during batch growth conditions (Soares Rodrigues, Wahl, and Gombert 2021).

The GO‐term “translational termination” encompasses 145 proteins, including components of the ribosome and elongation factors. For the observed sugar conditions 75 proteins were quantified, with up to 63 showing significant changes. For sucrose vs glucose, the highest odds ratio of 4.6 was observed with 63 changed protein levels, predominantly down regulations. The second most significant GO term, “cytoplasmic translation” is also linked to protein synthesis, and there is overlap with proteins of the “translation termination” term. Approximately 60 proteins were found to change with the carbon source in this category. Again, the highest odds ratio of 3.1 was observed for sucrose, while maltose showed a ratio of 2.1. In contrast, the fructose versus glucose comparison showed odds below 2, indicating smaller changes compared to the disaccharide conditions.

Additionally, for both fructose and sucrose, proteins of the category “response to heat” demonstrated altered levels, with eight proteins showing significant changes. In the case of maltose, the third most altered category was “cellular response to oxidative stress,” which was not observed for either fructose or sucrose.

These observations suggest that the response to fructose gradients was comparable to that of glucose, although with slightly lower levels of translation‐related proteins. This could be linked to the lower observed maximal uptake rate for fructose compared to glucose. At the same time, the reduced growth may have triggered an increase in stress‐related proteins included in the “response to heat” category.

The most significant changes were observed for sucrose, where many proteins of the cytosolic translational machinery of two GO categories showed a decreased expression. This reduction could be linked to the lower maximal uptake rate of sugars and in turn a lower maximal growth capacity requirement.

Maltose exhibited similar trends to sucrose, albeit less pronounced. Categories not present in the other conditions appeared to be linked to changes in redox metabolism, which may also correspond to the measured increased respiration under maltose conditions.

### 3.10 Changes in Glycolytic Proteins

**Figure 8:**
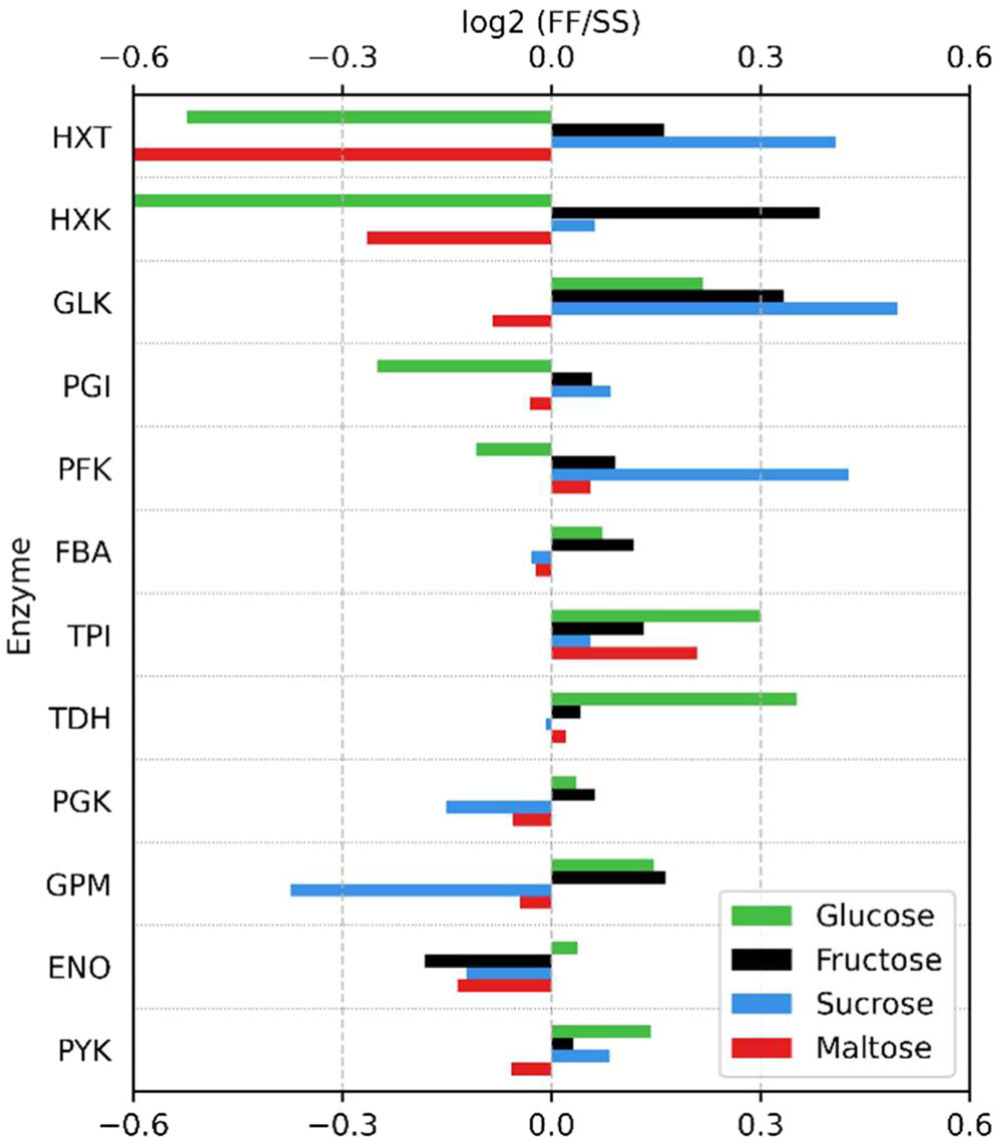
Protein concentration fold change (log2) between feast/famine and steady‐state chemostat conditions. Changes are shown for transporters as well as glycolytic enzymes.

Here, we focus on comparing the individual glycolytic enzymes involved in the initial steps of catabolism for the different sugar substrates. Modeling has indicated that at least two changes are required for a stable glycolytic operation under FF conditions: the upregulation lower glycolysis proteins (particularly triose phosphate isomerase, TDH) from steady‐state to FF conditions, combined with the downregulation of hexose transporters upper glycolytic proteins (Verhagen et al. Eerden, and Wahl 2022, 2023). These adaptations are essential to prevent a putative phosphate “deadlock” (van Heerden et al. 2014). Here the abundance to glucose uptake (HXT) and glucose phosphorylation (HXK) decreased by more than 30%. Lower glycolytic enzymes like triose phosphate isomerase (TPI) and TDH which are known to be abundant, increased by nearly 30%. Changes in protein concentrations downstream of TDH were less pronounced (Figure 8).

While the changes with maltose as a substrate partially followed those of glucose, this was not the case for fructose and sucrose as substrates (Figure 8). Instead, for fructose and sucrose, an upregulation of transporters and upper glycolytic proteins was observed, without a significant upregulation of lower glycolysis. The largest observed increase of glycolytic enzymes was observed for sucrose, with an increase of 41% for GLK (upper glycolysis). This inverse trend compared to glucose and maltose might be linked to the lower affinity of the fructose transporter, resulting in less significant changes in uptake flux. The upregulation of transporters was highest with sucrose as substrate, where glucose and fructose are transported over the membrane.

The distinct differences in protein adaptations observed for the four different substrates, in combination with the observed differences in uptake rates and especially metabolic response, highlights the differential regulation of central carbon metabolism under dynamic substrate conditions for these sugars. Previous research has demonstrated that the PKA/Ras signaling pathway is strongly activated by sucrose and glucose pulses, with an intermediate response to fructose, and no response to maltose. This differential activation triggered varied responses in the mobilization of storage carbohydrates and the induction and repression of associated genes (Botman et al. 2021). The observed adaptations in the proteome suggest the presence to additional substrate‐specific signaling mechanisms, allowing the cell to respond differently to repetitive sugar substrate pulses compared to continuous feeding with the same sugar. Monitoring the PKA/Ras signaling pathway during these transitions may provide valuable insights into the mechanisms underlying the differences in metabolic responses.

Furthermore, given that the CEN. PK113‐7D strain possesses a mutated CYR1 gene, investigating the metabolic responses to various sugar substrates in a strain without this mutation could clarify the involvement of the PKA pathway in regulating responses to repetitive sugar substrate pulses.

## 4 Conclusions and Outlook

The differential metabolic response of *S. cerevisiae* to various sugar substrates under dynamic conditions underscores the importance of considering the complexities of metabolic regulation in the design of bioprocesses. Although the sugar substrates are chemically very similar, they surprisingly elicited different metabolic responses under dynamic substrate conditions. This finding highlights the importance of studying a wider range of substrates, beyond glucose, to reflect diverse industrial feedstocks such as molasses, to better understand metabolic regulation in dynamic environments.

Especially with respect to metabolomics, proteomics, and fluxomics, several datasets are available for glucose, datasets for other carbon sources are sparse. Of special interest will be comprehensive datasets that include intracellular flux distributions. This should include flux data from ^13^C labeling experiments, in combination with kinetic modeling, which may assist with the evaluation of different hypotheses on the regulation of the metabolic response to dynamic conditions. Another challenge lies in the physiological differences between strains and substrate signaling. A comparative analysis with a non‐mutant CYR1 strain within a similar experimental setup would be beneficial. Additionally, understanding the impact of repetitive perturbations on production hosts will help to identify industrially relevant parameters that extend beyond biomass yield and respiration requirements.

## 5 Materials and Methods

### 5.1 Strain and Culture Conditions

The haploid yeast *S. cerevisiae* CEN. PK113‐7D, obtained from the *Centraalbureau van Schimmelcultures* (Fungal Biodiversity Center, Utrecht, The Netherlands), was used in this study. The cultivations were performed using a low‐salt Verduyn minimal medium (Canelas et al. 2009) with a fructose/glucose concentration of 7.5 g/L or a maltose/sucrose concentration of 7.12 g/L, with a feed of the same composition. 1L‐Erlenmeyer flasks containing 100 mL medium were inoculated with cells from a cryovial (glycerol, −80°C) and the inoculation cultures were subsequently grown for 10 h at 200 rpm and 30°C. The inoculation culture was used to inoculate a 7 L bioreactor (Applikon Biotechnology B.V., Delft, The Netherlands) containing a working volume of 4 L, controlled by a Biostat B Plus controller (Sartorius AG, Göttingen, Germany). The reactor was aerated with pressurized air at 1 L/min (0.25 vvm) using a Smart series mass flow controller 5850S (Brooks Instrument, Hatfield, PA, USA). The reactor was operated at 0.3 bar overpressure, at 30°C, with a stirrer speed of 600 rpm. The pH of the broth was maintained at 5.0 by automated addition of either 4 M KOH or 2 M H_2_SO_4_. Once the batch phase was completed upon the consumption of all glucose and produced overflow metabolites (indicated by a fast decrease in CO_2_ signal and a sharp increase in dissolved oxygen [DO]), the chemostat phase (steady‐state) was started at a dilution rate of 0.1 h^‐1^ for 50 h. DO was not controlled but observed to be between 65% to 70% during the steady‐state chemostat phase for the various cultivation on different sugar substrates. After about five residence times, sampling for extracellular and intracellular metabolites, biomass concentration, as well as proteomics was performed. This results in a total culture cultivation time of ~ 100 h, with 50 h of continuous feeding and 50 h with a block‐wise feeding regime. Extracellular and biomass samples were taken as technical triplicates, whereas proteomics samples were taken as technical duplicates.

### 5.2 Dynamic Feast Famine Setup

**Figure 9:**
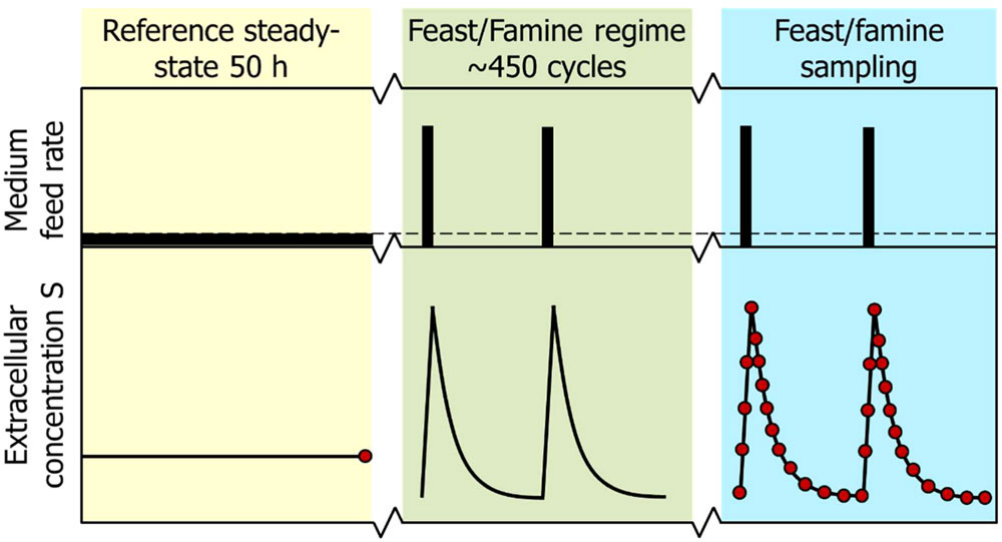
Profile of the experimental feeding regime. After a chemostat phase (reference steady‐state) of 50 h, a block‐wise feed is applied in a 400 s cycle at the same average substrate supply and dilution rate for another 50 h (adapted from Suarez‐Mendez et al. [2014]). At the top, a schematic overview of the feed rate during chemostat and feast/famine regimes is shown. At the bottom, the resulting extracellular substrate concentration profile in the fermentation broth is shown. Sampling time points are shown as red dots.

After five residence times (50 h) of continuous feeding, the feeding was changed to a block‐wise feeding regime, leading to a FF regime (Suarez‐Mendez et al. 2014). Cycles of 400 s were applied by a feeding medium for 20 s, followed by a period of 380 s of no feeding (Figure 9). The medium pump was controlled using an automatic timer (PTC‐1A, Programmable timing controller, Omega Engineering Inc., Stamford, CT, USA). During the 20‐second feeding period, 43 ± 1 mL of fresh medium were added. The same volume was subsequently withdrawn for 260 s at a flow rate of 0.166 ± 0.001 mL s^−1^ maintaining the broth volume nearly constant at 4 L. After about five residence times (450 cycles), sampling for extracellular and intracellular metabolites, biomass concentration as well as proteomics was performed.

### 5.3 Sample Acquisition and Analysis

#### 5.3.1 Extracellular Metabolites

For the analysis of extracellular metabolites, 1.5 mL broth was taken using a syringe containing ~ 26 g precooled (−20°C) stainless steel beads, which was subsequently filtered as described by Mashego et al. (Mashego et al. 2006). Extracellular acetate, ethanol, glucose and glycerol concentration were measured using HPLC or enzymatic assay, as described by Canelas et al. (Canelas et al. 2011). Biomass concentrations (cell dry weight) were determined using a gravimetrical method described by Suarez‐Mendez et al. (Suarez‐Mendez et al. 2014). The CO_2_ and O_2_ fractions in the off‐gas were determined using a combined infrared/paramagnetic NGA2000 analyzer (Rosemount Analytics, St. Louis, MO, USA).

#### 5.3.2 Intracellular Metabolites

Samples for the measurement of intracellular metabolites were taken by rapidly withdrawing 1 mL of broth and quenching it in 5 mL cold (−40°C) methanol, as described by Lange et al. and Canelas et al. (Canelas et al. 2009; Lange et al. 2001). Taken samples were weighted, and subsequently poured into a filtration setup (using a Supor‐200 cellulose membrane, 0.2 µm, 47 mm, Pall Corporation), which already contained 15 mL precooled (−40°C) methanol. After this, vacuum was applied, followed by the addition of 15 mL cold (−40°C) methanol to wash the biomass (Douma et al. 2010). The filter with the washed biomass was subsequently transferred to a 50 mL falcon tube containing 30 mL of a 75% (vol/vol) ethanol solution, preheated to 75°C. To this, 100 µL ^13^C yeast cell extract was added as internal standard (Wu et al. 2005). The tube was then shaken and put into a water bath at 95°C for 3 min to extract the intracellular metabolites. After extraction, the tubes were immediately cooled in an ice bath, and the filter was removed. The cell extract was subsequently stored at −80°C and later concentrated through complete evaporation of the aqueous ethanol solution and resuspended into 500 µL milliQ water, as described by Mashego et al. (Mashego et al. 2004). The resuspended samples were centrifuged at 15,000 g for 5 min at 1°C, and the supernatant was transferred to a new tube, which was subsequently centrifuged again to remove all solid components in the sample. The obtained supernatant was then transferred into a screw‐capped vial and stored at −80°C. Samples were analyzed by GC‐MS (Cipollina et al. 2009; Mashego et al. 2004; Wu et al. 2005) and LC‐MS (Seifar et al. 2009).

### 5.4 Proteomics Analysis

For each proteome sampling timepoint, two samples containing approximately 3 mg cell dry weight biomass were withdrawn into an Eppendorf tube and immediately centrifuged at 8000 g for 5 min at 4°C. Supernatant was discarded, the pellet was resuspended in 2 mL saline solution (0.9% NaCl) (cooled beforehand at 4°C) and centrifuged again (8000 g, 5 min, 4°C). The supernatant was discarded once more, again resuspended in 2 mL saline solution and centrifuged (8000 g, 5 min, 4°C). Then, the supernatant was discarded and the sample was snap‐frozen using liquid nitrogen and stored at −80°C until further analysis. For each timepoint, duplicate samples were taken. To process the samples for proteomics analysis, the cell mass was normalized to a dry weight of 1.6 mg and then mechanically lysed using 0.5‐mm zirconium beads and a PreCellys homogenizer. Proteins were isolated using Bligh and Dyer extraction (Sapcariu et al. 2014), followed by reduction, alkylation, and digestion using trypsin. The samples were analyzed in technical triplicates by liquid chromatography tandem mass spectrometry (LC‐MS/MS) using a Vanquish UHPLC coupled to a Q Exactive Plus Orbitrap MS (Thermo Fisher Scientific, Waltham, MA, USA). Peptides were separated using reverse‐phase chromatography using a gradient of water with 0.1% formic acid (solvent A) and acetonitrile with 0.1% formic acid (solvent B) from 2% B to 45% B in 50 min. Data‐dependent acquisition (DDA) was performed with a resolution setting at 70,000 within the 400‐ to 1600‐m/z range and a maximum injection time of 75 ms, followed by high‐energy collision‐induced dissociation activated (HCD) MS/MS on the top 15 most abundant precursors using a resolution setting of 17,500 and a 200‐ to 2000‐m/z range with a maximum injection time of 50 ms. The minimum intensity threshold for MS/MS was 1000 counts, and peptide species with 1 and > 8 charges were excluded. MS/MS spectra were analyzed with the SEQUEST HT search engine and Proteome Discoverer, version 2.3, against the proteins of *S. cerevisiae* (Uniprot, *S. cerevisiae* CEN. PK113‐7D, ID:UP000013192) (Nijkamp et al. 2012). Label‐free quantification was performed using the top three unique peptides measured for each protein. Retention time alignment was performed on the most abundant signals obtained from nonmodified peptides measured in all samples, and results were corrected for the total ion intensities measured for each sample. The data was analyzed for statistical differences using Perseus 1.6.10.45 (Tyanova et al. 2016). A two‐sample test was used to determine the significance of the fold change, with a significance level threshold of *p* < 0.01, and at least two unique peptides per protein. Pathway enrichment analysis was performed according to Schnessner et al. (2022). The enrichment analysis is run using a Fisher's exact test, including multiple hypothesis correction, with a false detection rate (FDR) of 1%.

## Supporting information

Supporting information.

## Data Availability

The dataset on proteome fold changes between continuous feeding and block‐wise feeding conditions, analyzed in this study, can be found in the 4TU. ResearchData repository at https://doi.org/10.4121/19008833 and https://doi.org/10.4121/21541416. The analyzed metabolomics dataset for continuous feeding and blockwise feeding conditions can be found in the same repository at https://doi.org/10.4121/21692057. The analyzed glucose dataset generated by Suarez‐Mendez et al. (2014), consisting of extracellular and intracellular metabolite data, as well as biomass concentrations, can be found at https://doi.org/10.3390/metabo4020347.
