# Supplementary material for: A Dive Into Yeast's Sugar Diet—Comparing the Metabolic Response of Glucose, Fructose, Sucrose, and Maltose Under Dynamic Feast/Famine Conditions
Source: Biotechnol Bioeng. 2025 Jan 26;122(4):1035–50. doi: 10.1002/bit.28935 (PMC11895419; doi:10.1002/bit.28935)
Supplement: Supplementary file 1 — Supporting information. [file BIT-122-1035-s001.docx]

**Appendix**

**A dive into yeast’s sugar diet – Comparing the metabolic response of glucose, fructose, sucrose & maltose under dynamic feast/famine conditions**

Koen J. A. Verhagen^1,#^, Ilse H. Pardijs^1,+^, Hendrik Matthijs van Klaveren^1^ and Sebastian Aljoscha Wahl^1,*^

**Figure A1.** Concentration measurement of intracellular glycolytic metabolites during a 400s feast/famine cycle for glucose (green), fructose (black), sucrose (blue) and maltose (red). Dashed lines indicate the steady state level for each metabolite for the different sugars respectively. Data for the glucose cultivation conditions was generated by Suarez-Mendez et al. (2014), while data for the fructose, sucrose and maltose cultivation conditions was generated in this study.

**Figure A2.** Concentration measurement of intracellular nucleotides during a 400s feast/famine cycle for glucose (green), fructose (black), sucrose (blue) and maltose (red). Dashed lines indicate the steady state level for each metabolite for the different sugars respectively. Data for the glucose cultivation conditions was generated by Suarez-Mendez et al. (2014), while data for the fructose, sucrose and maltose cultivation conditions was generated in this study.

**Figure A3.** Concentration measurement of intracellular metabolites of the pentose phosphate pathway during a 400s feast/famine cycle for glucose (green), fructose (black), sucrose (blue) and maltose (red). Dashed lines indicate the steady state level for each metabolite for the different sugars respectively. Data for the glucose cultivation conditions was generated by Suarez-Mendez et al. (2014), while data for the fructose, sucrose and maltose cultivation conditions was generated in this study.

**Figure A4.** Concentration measurement of intracellular metabolites of the TCA cycle during a 400s feast/famine cycle for glucose (green), fructose (black), sucrose (blue) and maltose (red). Dashed lines indicate the steady state level for each metabolite for the different sugars respectively. Data for the glucose cultivation conditions was generated by Suarez-Mendez et al. (2014), while data for the fructose, sucrose and maltose cultivation conditions was generated in this study.

**Figure A2.** Concentration measurement of intracellular nucleotides during a 400s feast/famine cycle for glucose (green), fructose (black), sucrose (blue) and maltose (red). Dashed lines indicate the steady state level for each metabolite for the different sugars respectively.

**Figure A5.** Concentration measurement of intracellular metabolites of the trehalose cycle during a 400s feast/famine cycle for glucose (green), fructose (black), sucrose (blue) and maltose (red). Dashed lines indicate the steady state level for each metabolite for the different sugars respectively. Data for the glucose cultivation conditions was generated by Suarez-Mendez et al. (2014), while data for the fructose, sucrose and maltose cultivation conditions was generated in this study..
